# Fluence smoothing evaluation for whole‐breast automatically generated treatment plans

**DOI:** 10.1002/acm2.14564

**Published:** 2024-11-29

**Authors:** Giulianne Rivelli R. Zaratim, Luis Felipe Oliveira e Silva, Ricardo G. dos Reis, Cristiano Jacques M. R. Mendes, Marília Miranda F. Gomes

**Affiliations:** ^1^ Department of Biomedical Engineering University of Brasilia Brasília Brazil; ^2^ CONFIAR Radiotherapy Goiânia Goiás Brazil; ^3^ Department of Radiation Oncology University Hospital of Brasília Brasília Federal District Brazil

**Keywords:** automated treatment planning, breast cancer, complexity, fluence smoothing, IMRT

## Abstract

**Purpose:**

This study aimed to identify the fluence smoothing threshold that preserves the dosimetric quality of planning for breast cancer intensity‐modulated radiation therapy (IMRT).

**Material and methods:**

We conducted automated treatment planning for 60 breast cancer patients using the Eclipse Scripting Application Programming Interface. The plans included four‐field IMRT, emphasizing smoothing weight combinations while maintaining a 4:3 aspect ratio between the X and Y directions. Four weight sets (40 × 30, 100 × 75, 150 × 115.2, and 200 × 150) were tested, resulting in four plans per patient. A total dose of 40.05 Gy over 15 fractions was prescribed. Optimization weigths were dynamically adjusted based on dosimetric evaluations, with the maximum coverage priority set at 200. Statistical analyses were used to assess the dosimetric data.

**Results:**

The median planning target volume (PTV) coverage varied across smoothing levels, with default smoothing (40 × 30) providing superior median PTV coverage. Lung constraints showed significant differences mainly at higher smoothing levels. Heart constraints exhibited less variation between smoothing levels, with significant differences primarily in the maximum and mean doses for right‐sided patients and between default and higher smoothing levels for left‐sided patients. No significant differences were observed in contralateral breast constraints among all smoothing levels, except at the maximum level for right‐sided patients. Monitor units decreased with increasing smoothing weight, showing significant differences between default and other settings. For right‐sided patients, the median number of monitor units varied from 1346 (40 × 30) to 754 (200 × 150), and for left‐sided patients, from 1333 (40 × 30) to 804 (200 × 150). Chi‐square tests revealed differences in dose constraint adherence between default and maximum smoothing levels, particularly in target coverage.

**Conclusion:**

Our findings suggest that using a ratio of smoothing weights to target priorities between 1:1.5 and 1:1.6 leads to a favorable balance between complexity and dosimetric plan quality, with no significant impacts on dose constraint adherence.

## 1 INTRODUCTION

Intensity‐modulated radiation therapy (IMRT) constitutes a commonly used technique capable of producing absorbed‐dose distributions that excel those achieved by 3D conformal radiation therapy treatments in numerous circumstances.^1^ The use of modulated intensity maps can enhance planning by generating dose distributions that conform closely to the shape of the tumor. Conversely, complex fluence maps can result in dosimetric inaccuracies, inefficient plan delivery,^2^ and a greater number of monitor units (MUs) for treatment execution.

In this context, the implementation of fluence smoothing techniques allows the regulation of fluence complexity, thus resulting in a diminished requirement for MUs. Nevertheless, this smoothing process may influence the ultimate dose distribution, particularly in areas requiring steeper dose gradients.^3^ As a result, it is essential to carefully evaluate the trade‐offs between the complexity of the plan and the dosimetric quality of the treatment plan.^2^

In certain optimization algorithms, it is possible to adjust the degree of modulation by manipulating the smoothing levels of leaf motion in both the X direction and the Y direction. In such methods, the cost function includes terms related to the smoothing levels.^4^ Additionally, they use weights that correspond to both the importance of absorbed doses for the target and organs at risk (OAR) and to the degree of smoothness applied to each IMRT beam.

Another aspect to consider, within the scope of research and clinical practice, is the importance of implementing automated plans for dosimetric comparisons. This approach stands as a crucial tool for mitigating potential biases inherent to purely manual planning techniques.^5^, ^6^, ^7^ The objective of this study was to assess the variations in dose distribution among treatment plans for breast IMRT that are automatically generated, each with different smoothing values. Through this investigation, we aimed to elucidate the impact of smoothing parameters on dose distribution, thereby contributing valuable insights to the optimization and refinement of IMRT treatment planning strategies.

## 2 MATERIAL AND METHODS

### 2.1 Patient selection

In this study, automated treatment plans were generated via the Eclipse Scripting Application Programming Interface (ESAPI). These plans were designed for 30 breast cancer patients per laterality (right and left), totaling 60 patients. The research was conducted in anonymized patient data.

### 2.2 Contouring and volume definitions

The organ contours adhered to the Breast Cancer Atlas guidelines by the Radiation Therapy Oncology Group (RTOG),^8^ while the planning target volume (PTV) included the clinical target volume (CTV), comprising the entire breast, with an additional 5 mm margin. For the optimization process, the optimization PTV was generated by cropping the PTV 5 mm from the skin surface. All contours were thoroughly reviewed by a radiation oncologist.

### 2.3 Treatment planning

We adhered to the RTOG 1005 treatment protocol, specifically to its Arm II, prescribing a dose of 40.05 Gy across 15 fractions.^9^ The computed tomography (CT) scans were conducted with patients in the head‐first supine position on a breast board, with arms positioned overhead during free‐breathing. For treatment planning, we used the Eclipse treatment planning system by Varian Medical Systems (Palo Alto, California, USA), version 16, alongside the Halcyon 3.0 treatment engine, which is also from Varian Medical Systems.

With respect to dose calculations, we used the analytical anisotropic algorithm (AAA) version 16.1, whereas for leaf motion calculations, we used the Smart Leaf Motion Calculator (SMLC) version 16.1, and for optimization, we used the Photon Optimizer (PO) version 16.1. The calculation and optimization grid used in the study is 2.5 mm. The skin flash was applied with a 1.5 cm expansion, utilizing the fluence of pixels within 0.5 cm from the surface.

The beam arrangement employed four‐field IMRT, including tangent fields with a 10‐degree angled field each, oriented clockwise for the left breast and counterclockwise for the right breast. All beams were set with a 6 MV‐FFF energy with the sliding window dose delivery technique. The gantry angles for the tangent fields were chosen as described in previous studies.^10^, ^11^ In this approach, we use the gantry angle that generates the minimum intersection area between the PTV and the ipsilateral lung within a range of angles in the Beam's Eye View projections. The auxiliary fields were angled from these tangent beams and the collimator was set at zero degrees.

### 2.4 Smoothing weight combinations

According to the manufacturer,^12^ the optimal fluence must be smooth to facilitate leaf motion calculations using the leaf motion calculator. This smoothness is achieved by incorporating an objective that accounts for the differences between adjacent fluence values in both the X and Y directions. Within a target projection, the smoothing cost is related to the squared curvature of the fluence profile in both the X direction and the Y direction. At the boundaries of the target projection, only the increase in fluence toward the exterior incurs a cost, allowing the fluence to conform to and decrease at the edges without additional cost.

To investigate the impact of various combinations of smoothing weights on the treatment plans, a fixed aspect ratio of 4:3 between the X and Y directions was maintained throughout the strategies. This fixed 4:3 ratio adheres to the vendor's default smoothing settings,^12^ while allowing for variation in the weights assigned to the desired values. Four distinct sets of smoothing weights were utilized, corresponding to X‐axis values of 40, 100, 150, and 200. These values correspond to the priorities assigned to each smoothing parameter in the cost function within the optimization process. This led to four automatically generated treatment plans for each patient. During the optimization process, the initial weight assigned to the coverage optimization criteria (D95 ≥ 4005 cGy) was capped at a maximum of 120.

A previous study described and evaluated the automation algorithm we used for treatment planning.^11^ The plan automatically sets the beams and optimization objectives. After the dose calculation, the algorithm assesses compliance with the dose constraints outlined in the RTOG 1005 guidelines. If any objective is not met, a subsequent optimization is executed, involving adjustments to the priority settings (objective weights). A subsequent evaluation was performed after AAA dose calculation. These adjustments are guided by the discrepancies observed between the dose constraints defined in RTOG 1005 and the doses computed by the AAA algorithm. This entire procedure is repeated once more, albeit with a focus on refining the doses specified within the optimization objectives. In the optimization process, we abstained from the use of Intermediate Dose tool. For this study, we limited the adjustments to 200 for lower objective target priority.

Following planning and prior to automatic skin flash addition, all plans with smooth weights greater than the default weight underwent normalization to ensure that the volume of the PTV that received 100% of the dose was the same in all plans.

### 2.5 Analysis

We evaluated the adherence of the plans to the protocol‐established dose tolerances according to three categories, namely ideal, acceptable, and unacceptable, as shown in Table 1.

**TABLE 1:** Dose constraints according to the RTOG 1005 protocol.^9^

	Ideal	Acceptable
PTV	≥95% of the breast PTV Eval will receive ≥95% of the prescribed dose	≥90% of the breast PTV Eval will receive ≥90% of the prescribed dose
	Dmax is ≤115% of the prescribed dose	Dmax is ≤120% of the prescribed dose
Heart	≤5% of the heart for left‐sided cancer ≤16 Gy 0% of the heart for right‐sided receives ≤16 Gy	≤5% of the heart for left‐sided cancer ≤20 Gy 0% of the heart for right‐sided receives ≤20 Gy
	≤30% of the heart for left‐sided cancer ≥8 Gy ≤10% of the heart for right‐sided receives ≥8 Gy	≤35% of the heart for left‐sided cancer ≥8 Gy ≤15% of the heart for right‐sided receives ≥8 Gy
	Mean dose is ≤400 cGy	Mean dose is ≤320 cGy
Ipsilateral lung	≤15% of the ipsilateral lung receives ≥16 Gy	≤20% of the ipsilateral lung receives ≥16 Gy
	≤35% of the ipsilateral lung receives ≥8 Gy	≤40% of the ipsilateral lung receives ≥8 Gy
	≤50% of the ipsilateral lung receives ≥4 Gy	≤55% of the ipsilateral lung receives ≥4 Gy
Contralateral lung	≤10% receives 4 Gy	≤15% receives 4 Gy
Contralateral breast	Dmax is ≤240 cGy	Dmax is ≤264 cGy

To assess the significance of the findings derived from these automated plans, we conducted statistical analyses. These analyses involved the use of Wilcoxon tests and chi‐square tests for proportions, both at a significance level of 0.05. They enabled comparisons of the impact of various smoothing weight combinations on dosimetric data and the number of monitor units. For these statistical analyses, we used the SciPy library in the Python programming language.^13^ Moreover, to validate the dose distribution calculations, an independent verification was performed via ClearCalc software (Radformation Inc., New York, USA). ClearCalc software employs a finite‐size pencil beam algorithm for the independent verification of calculated doses in clinical treatment plans.^14^ In our study, we assessed the discrepancy between the dose calculated by the treatment planning system and the dose calculated by ClearCalc at a point near the isocenter, maintaining a minimum distance of 2 cm from the chest wall.

## 3 RESULTS

The dosimetric data of the automatic treatment plans generated with different smoothing weights for the breast cancer patients are shown in Table 2. The results of the Wilcoxon tests are also presented in Table 2. Since most of the values of the contralateral lung RTOG 1005 constraint (V4Gy) are zero, the comparison of this dosimetric parameter was omitted.

**TABLE 2:** Median values and interquartile ranges of dosimetric parameters for our cohort of 60 patients for whole‐breast irradiation and *p*‐values from the Wilcoxon test between the various smoothing levels compared to the default smoothing level for each dosimetric parameter.

			Dose constraints									
			PTV			Heart			Ipsilateral lung			Contralateral breast
Side	Smooth (X,Y)		V95 (%)	V90 (%)	D_max_ (%)	D_max_/ D5%^a^ (Gy)	V8Gy (%)	D_mean_ (Gy)	V16Gy (%)	V8Gy (%)	V4Gy (%)	D_max_ (Gy)
Right	40 × 30	Median (IQR)	96.64 (3.30)	98.21 (2.83)	107.24 (1.16)	4.28 (1.04)	0.00 (0.00)	1.10 (0.20)	11.72 (2.38)	18.12 (4.96)	31.31 (5.20)	3.60 (11.05)
	100 × 75	Median (IQR)	96.14 (2.95)	97.97 (2.41)	106.45 (0.59)	4.30 (1.04)	0.00 (0.00)	1.10 0.22)	11.90 (2.02)	18.59 (4.11)	31.89 (4.45)	4.86 (14.15)
		*p*	<0.01	<0.01	<0.01	0.80	0.59	<0.01	<0.01	0.04	<0.01	0.58
	150 × 112.5	Median (IQR)	95.38 (2.09)	97.32 (1.93)	106.67 (0.36)	4.35 (1.10)	0.00 (0.00)	1.12 (0.22)	12.30 (1.87)	19.60 (3.67)	33.26 (4.90)	5.22 (12.86)
		*p*	<0.01	<0.01	<0.01	<0.01	0.11	<0.01	<0.01	<0.01	<0.01	0.83
	200 × 150	Median (IQR)	94.74 (1.39)	96.88 (1.76)	107.06 (0.57)	4.49 (1.30)	0.00 (0.00)	1.15 (0.24)	13.27 (2.19)	21.12 (4.02)	34.46 (5.62)	7.93 (13.13)
		*p*	<0.01	<0.01	0.03	<0.01	0.07	<0.01	<0.01	<0.01	<0.01	0.03
Left	40 × 30	Median (IQR)	95.40 (2.33)	96.65 (2.18)	108.23 (1.84)	8.76 (3.06)	5.51 (2.38)	2.78 (0.37)	11.03 (3.31)	17.69 (4.72)	31.49 (6.93)	3.07 (14.97)
	100 × 75	Median (IQR)	94.72 (2.20)	96.30 (2.23)	106.47 (0.92)	8.97 (3.04)	5.58 (2.26)	2.77 (0.36)	11.18 (2.81)	17.79 (3.92)	31.44 (6.37)	3.97 (14.65)
		*p*	<0.01	<0.01	<0.01	0.19	0.93	0.18	<0.01	0.08	0.44	0.80
	150 × 112.5	Median (IQR)	94.09 (2.05)	95.84 (1.87)	106.76 (0.45)	9.12 (2.11)	5.67 (1.50)	2.82 (0.34)	11.73 (2.26)	19.14 (3.19)	32.17 (5.80)	5.01 (15.95)
		***p***	<0.01	<0.01	<0.01	0.77	0.23	0.69	<0.01	<0.01	<0.01	0.63
	200 × 150	Median (IQR)	93.77 (1.86)	95.32 (1.44)	107.06 (0.56)	9.22 (2.04)	5.94 (1.40)	2.87 (0.36)	12.43 (2.75)	20.53 (3.20)	33.61 (5.51)	5.94 (16.33)
		*p*	<0.01	<0.01	<0.01	0.05	0.04	<0.01	<0.01	<0.01	<0.01	0.45

^a^ Dmax refers to the dose constraint for right‐sided patients, and D5% refers to the dose constraint for left‐sided patients.

The obtained p‐values indicate that there are statistically significant differences in the distributions of PTV coverage parameters when the default smoothing level is compared with all other smoothing levels, even after normalization. Furthermore, as illustrated by the dosimetric data in Table 2, the results indicate that the median PTV V95 and V90 values of the default smoothing level surpass those of the other levels. Conversely, the maximum PTV dose, which also exhibited statistical significance, presented a greater median value for the default smoothing level (40 × 30), and a lower median value for the 100 × 75 smoothing level.

With respect to ipsilateral lung constraints, we observed statistically significant differences in all comparisons for the two highest smoothing levels, but not always for the 100 × 75 smoothing level. Regarding heart constraints, fewer statistically significant differences were observed between the various smoothing levels; statistical significance was primarily detected in the maximum dose (D_max_) for the two highest smoothing levels and in the mean dose (D_mean_) for all smoothing levels for the right‐sided patients. For the left‐sided patients, there were statistically significant differences between the default smoothing level and the higher smoothing level for the V8 and the mean heart dose. Notably, no statistically significant differences were identified in the dose distributions pertaining to contralateral breast constraint across all the examined smoothing levels, except for the higher smoothing level for right‐sided patients.

**FIGURE 1:**
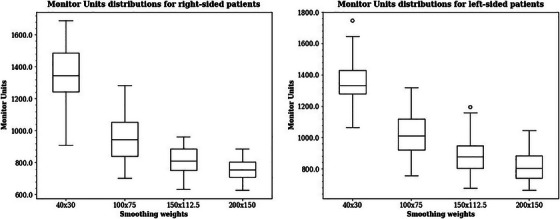
Monitor unit distributions for whole‐breast irradiation patients.

There was a statistically significant difference between the number of monitor units at the default smoothing level (40 × 30) and the other smoothing settings (*p*‐values < 0.01). The median number of monitor units decreased with increasing smoothing weight. We also evaluated the MU factor, defined as the ratio between the monitor units and the fraction dose. For right‐sided patients, the median MUs were 1346 (MU factor = 5.04) for the 40 × 30 smooth, 945 (MU factor = 3.54) for the 100 × 75 smooth, 812 (MU factor = 3.04) for 150 × 112.5 smooth and 754 (MU factor = 2.82) for the 200 × 150 smooth. For left‐sided patients, the median MUs were 1333 (MU factor = 4.99) for the 40 × 30 smooth, 1013 (MU factor = 3.79) for the 100 × 75 smooth, 879 (MU factor = 3.29) for the 150 × 112.5 smooth, and 804 (MU factor = 3.01) for the 200 × 150 smooth. The MUs described were tabulated subsequent to the automated application of the skin flash. These distributions are depicted in the boxplots shown in Figure 1.

**FIGURE 2:**
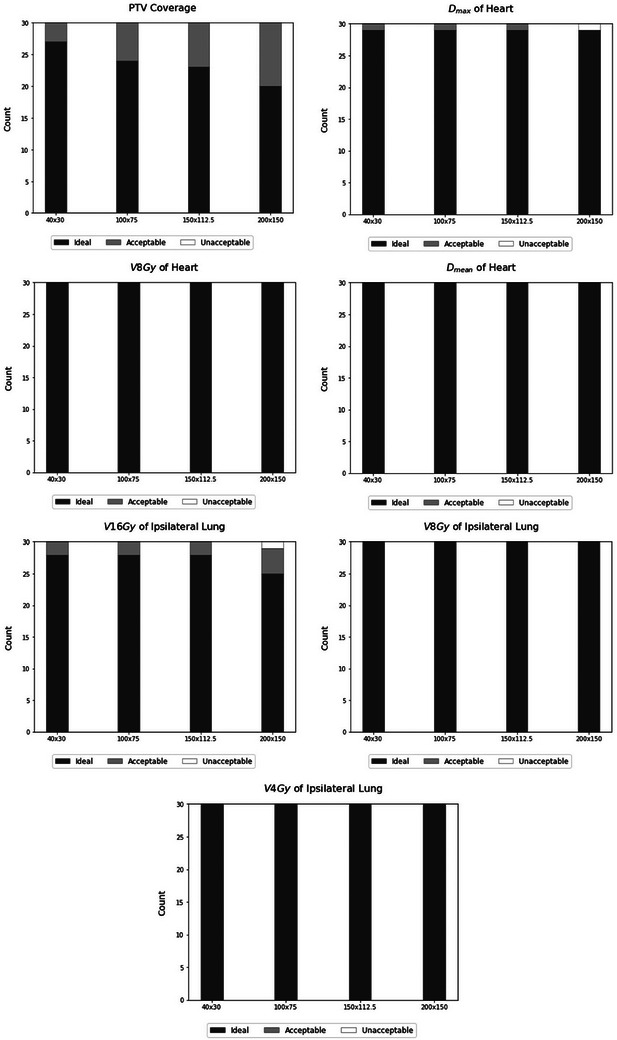
Number of plans meeting the dose constraints for right‐sided patients.

**FIGURE 3:**
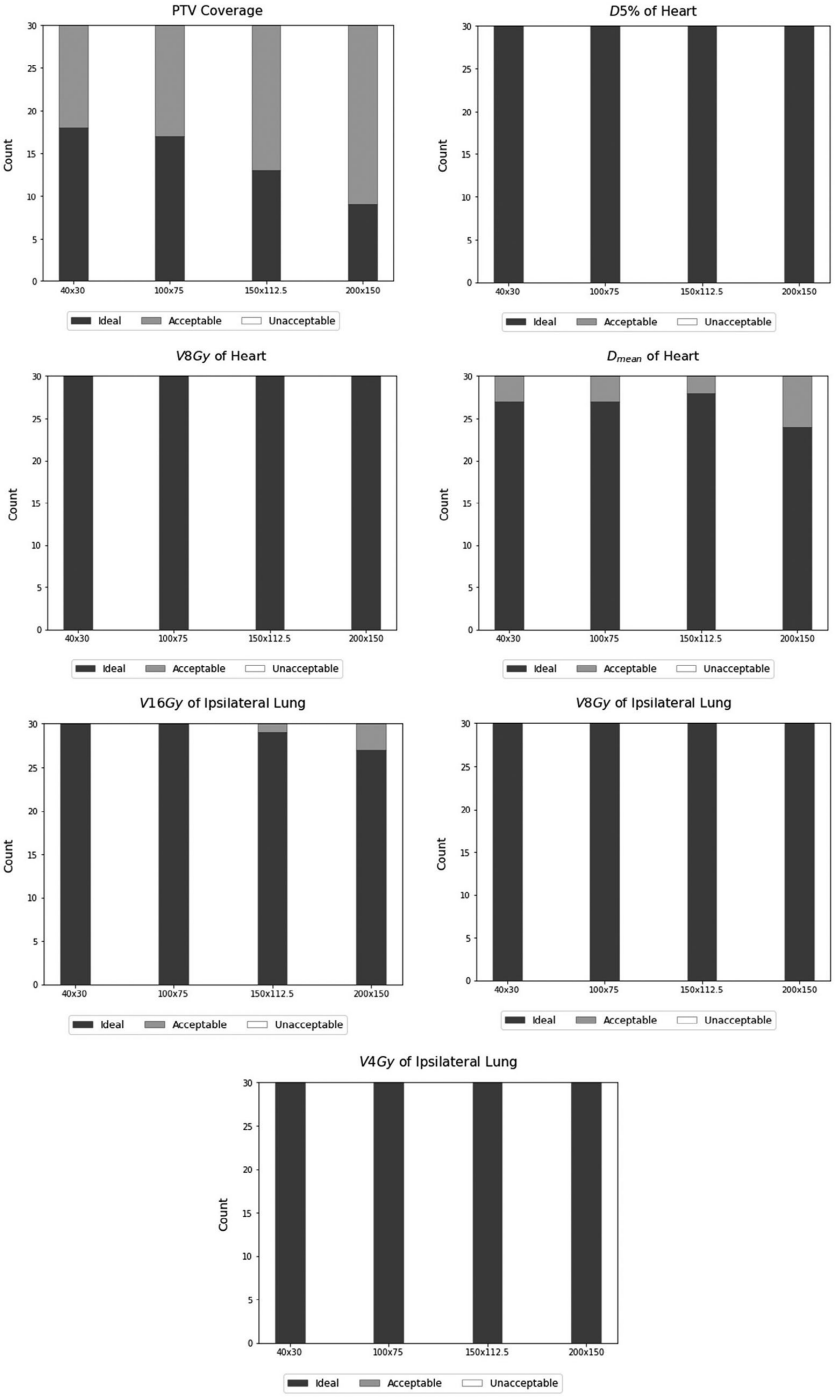
Number of plans meeting the dose constraints for left‐sided patients.

The results for the number of plans meeting the dose constraints, categorized as ideal, acceptable, and unacceptable dose parameters, are depicted in Figures 2 and 3 for right and left‐sided patients, respectively. To evaluate potential disparities in the number of plans meeting dose constraints, we used the chi‐squared test. We identified a statistically significant difference solely between the ideal target coverage when comparing the default smoothing weight (40 × 30) and the maximum smoothing weight (200 × 150) for left‐sided and right‐sided patients (*p*‐values of 0.02 and 0.03, respectively).

The maximum weights assigned to PTV coverage requirement (D95 ≥ 4005 cGy) following dynamic optimization are collated and presented as the mean maximum weights with their respective standard deviations. Additionally, the average relative weights were assessed and are displayed in Table 3.

**TABLE 3:** The mean values of the coverage weights for the D95 ≥4005 cGy requirement are presented as the mean value ± standard deviation (minimum‐maximum value).

	Side							
	Right				Left			
	40 × 30	100 × 75	150 × 112.5	200 × 150	40 × 30	100 × 75	150 × 112.5	200 × 150
Mean target coverage weights	152 ± 31 (120–200)	152 ± 31 (120–200)	159 ± 29 (124–200)	173 ± 2 (137–200)	156 ± 29 (120–200)	157 ± 29 (120–200)	167 ± 28 (120–200)	183 ± 21 (137–200)
Ratio	1:3.8	1:1.5	1:1.1	1:0.9	1:3.9	1:1.6	1:1.1	1:0.9

The ratio between these values and the smoothing weight is also presented.

The results of the independent calculations showed a strong agreement with the treatment planning system calculations. All plans exhibited a deviation of less than 3% between the different planning strategies. The mean percentage dose differences between the Treatment Planning System and the ClearCalc were 2.71 ± 0.58%, 2.68 ± 0.57%, 2.69 ± 0.56%, and 2.69 ± 0.55% for the 40 × 30, 100 × 75, 150 × 112.5, and 200 × 150 smoothing priorities for left‐sided patients, respectively. For right‐sided patients, the mean percentage dose differences were 2.79 ± 0.40%, 2.76 ± 0.44%, 2.77 ± 0.41%, and 2.79 ± 0.40% for the 40 × 30, 100 × 75, 150 × 112.5, and 200 × 150 smoothing priorities, respectively.

## 4 DISCUSSION

The widespread use of intensity‐modulated radiation therapy in radiation oncology underscores the importance of a critical evaluation of its complexity, considering potential dosimetric effects in tumor sites that move during irradiation. When a dynamic multileaf collimator is used to conform the beam, physicists should consider the possibility of dose blurring and the interplay effect.^15^, ^16^ There is also a concern when considering possible patient setup errors during treatment. It has been discussed that increased plan complexity may result in a more pronounced degradation of plan quality in instances where patient setup errors occur throughout the course of treatment, particularly compared with simpler treatment modalities.^17^ Furthermore, the potential increases in treatment duration, due to increased numbers of monitor units, require careful consideration. This is important since minimizing delivery time while maintaining treatment quality can reduce intrafractional patient motion and increase the spatial accuracy of treatment delivery.^18^ These factors may contribute to the preference among many physicists for less complex treatment plans in such cases.

Research conducted by Craft et al.^19^ indicates the presence of a critical level of complexity required to attain satisfactory plans, yet additional complexity beyond this threshold does not improve plan quality and results in treatments that require considerably more time for delivery. Thus, it is important to bear in mind that a high level of plan complexity does not necessarily correlate with superior plan quality.

Additionally, certain physical factors may impact how physicists manage treatment planning. In a study evaluating the dosimetric and radiobiological impact of the flattening filter free (FFF) beam and calculation algorithms, Manna et al.^20^ concluded that the FFF beam implies a significant increase in MUs compared with the flattened beam. This finding is consistent with several other studies comparing MU requirements for plans generated using FFF beams versus flattened beams, all of which consistently reported an increase in MU with FFF beams.^21^, ^22^, ^23^, ^24^ Thus, in accelerators such as Halcyon, which do not have a flattening filter, methods that potentially reduce the number of MUs should be considered.

One of the major concerns regarding methods to reduce complexity, such as smoothing procedures, is that these methods commonly exchange target coverage or tissue sparing for delivery efficiency.^25^, ^26^ Therefore, it is recommended that the application of these methods be accompanied by a careful evaluation of the trade‐off between the complexity and dosimetric quality of the treatment plan.^2^ In the approach described in this work, our primary objective is to explore the smoothing penalization threshold within the cost function that does not compromise the dosimetric quality of the treatment plan.

Notably, several alternative metrics for assessing IMRT complexity have been presented in the literature.^27^, ^28^ While some correlations between these metrics exist,^29^ Kamperis et al.^25^ commented that the existence of so many might be attributed to the fact that a singular metric may be insufficient in capturing the different aspects of a plan's modulation degree, and it may not be universally suitable for all treatment planning systems. However, a recent study that evaluated the complexity of IMRT treatment plans for breast cancer patients revealed that for linear accelerators, the most appropriate complexity metric was the MU factor.^30^

The literature shows that the use of IMRT can lead to an approximately twofold increase in the occurrence of radiation‐induced carcinomas in contrast to conventional radiation therapy. As Hall and Wuu^31^, ^32^ noted, this phenomenon can be attributed to several factors. First, the dose distribution in IMRT typically results in a larger volume exposed to lower radiation doses. Second, IMRT tends to require a greater number of monitor units than conventional radiation therapy does. Thus, despite its apparent simplicity,^25^ the assessment of the number of monitor units remains a valuable metric within the field of radiobiology.

In their work, Kamperis et al.^25^ discuss that the best way to reduce complexity resides within the planning cost function itself. According to their viewpoint, introducing penalties for beam modulation within the objective function can effectively reduce complexity while simultaneously preserving dosimetric quality.^25^, ^26^, ^33^, ^34^ Thus, in alignment with their insights, the adjustment of the modulation degree through the manipulation of smoothing levels governing leaf motion in both the X and Y directions, as employed in our investigation, emerges as an assertive choice.

To the best of our knowledge, very few studies in the literature have evaluated the dosimetric impact of variations in fluence smoothing, and studies employing automatic treatment planning to ensure unbiased planning are lacking.

Saroj et al.^35^ investigated the reduction in complexity through the alteration of fluence smoothing in reoptimized cervix cancer treatment plans, incorporating various weights in the X and Y directions. In their study, organ‐at‐risk dose variations were not significant with increasing smoothing. Moreover, the number of monitor units decreased with increased smoothing, accompanied by a reduction in the number of treatment segments. The authors recommend the use of smoothing levels of at least 80 in both the X and Y directions to achieve optimal treatment plan doses for the PTV and OARs, along with reduced monitor units. The same replanning methodology was implemented by Niyas et al.^36^ in their investigation of the impact of smoothing in patients with nasopharyngeal and lung cancers. The findings echo those of the aforementioned study, leading to the recommendation of employing smoothing levels up to X = 70 and Y = 60, particularly for nasopharyngeal and lung patients, without any considerable changes in OAR doses or improvements in the deliverability of the plans.

In research conducted by Anker et al.,^4^ the group evaluated the fluence smoothing functionality across three different treatment planning systems, including Eclipse, for head and neck cancers, glioblastomas, and prostate cases. They recommend maintaining structure‐dose‐priority weights within the range of 0–100 and utilizing vendor‐recommended values for (X, Y) smoothing during the initial planning stages. They subsequently propose adjusting the smoothing levels within the intervals of (X = 40–80) and (Y = 30–60) post optimization to explore potential benefits. Notably, a plan without smoothing required 180% more monitor units for delivery.

Our study indicates that the default smoothing level (40 × 30) in treatment planning generally enhances PTV coverage, achieving higher median PTV doses than other levels. Although these differences were statistically significant, their magnitude was small and likely resulted in similar probabilities of disease control, given that the maximum median difference was 1.9%. Significant differences in lung constraints were mainly observed at the highest smoothing levels, reaching up to a 3.1% median difference in V4, whereas this trend was not consistent at the 100 × 75 setting, which had a maximum median difference of 0.58% in V4. Heart constraints varied less significantly across smoothing levels, with the main difference observed for V8 (0.43%) when the 40 × 30 and 200 × 150 smoothing levels were compared.

The study also revealed a decrease in median monitor unit counts with increased smoothing, which was consistent for both right‐ and left‐sided patients. Notably, the number of plans meeting the dose constraints for target coverage at the default level was similar to that at the 100 × 75 and 150 × 112.5 levels, suggesting a possible threshold for smoothing efficacy. By comparing the default smoothing level (40 × 30) with the maximum smoothing weight (200 × 150), our findings revealed differences between right‐ and left‐sided patients. For both sides, a statistically significant difference was observed in target coverage. Notably, there was one instance where the V16Gy for the ipsilateral lung and one where the maximum heart dose were deemed unacceptable for right‐sided patients at the maximum smoothing level.

When conducting a trade‐off between complexity and the reduction of dosimetric plan quality, our findings suggest that employing a 100 × 75 smoothing level is the optimal choice, as it has no statistically significant impact on dose constraint approval. Furthermore, although additional statistically significant differences were observed between the default smoothing and the 150 × 112.5 smoothing level, there were no significant disparities in the dose constraint approval rates. However, upon assessment of the maximum smoothing level (200 × 150), plans start to show significant decreases in coverage parameters and an increase in dose to OAR. When comparing the ratios between the smoothing weights and the priorities assigned to the target, we observe that the optimal value falls within the range of 1:1.5 to 1:1.6. Plan quality decreases as the ratio approaches 1:1. This insight is pivotal, as it is applicable to any planner and is not contingent upon a specific set of optimization weights.

The planning strategy employed in this study aligns with the institution's standard approach. Traditionally, the smoothing component's weight is determined at the outset of the planning process and remains unchanged during optimizations, regardless of potential increases in other requirement weights. In future research, a dynamic adjustment of smoothing weights proportional to the elevation of target priority could be considered to assess whether dosimetric outcomes surpass those achieved with the current approach.

## 5 CONCLUSION

The study suggested that, within the parameters we investigated, the optimal balance between complexity and dosimetric plan quality can be achieved with a smoothing level of 100 × 75. Specifically, when the ratios between smoothing weights and target priorities are examined, the ideal value falls within the range of 1:1.5 to 1:1.6. In our sample, this ratio successfully met the dose constraints without significantly impacting plan quality. Notably, we observed a decline in plan quality as the ratio approached 1:1.

## AUTHOR CONTRIBUTIONS

**Giulianne Rivelli R. Zaratim**: Conceptualization; data curation; formal analysis; investigation; methodology; software; writing—original draft; writing—review & editing. **Luis Felipe Oliveira e Silva**: Conceptualization; data curation; formal analysis; investigation; methodology; software; writing—original draft; writing—review & editing. **Ricardo G. dos Reis**: Conceptualization; data curation; formal analysis; investigation; methodology; writing—original draft; writing—review & editing. **Cristiano Jacques M. R. Mendes**: Supervision; writing—original draft; writing—review & editing. **Marilia Miranda F. Gomes**: Project administration; supervision; writing—original draft; writing—review & editing.

## CONFLICT OF INTEREST STATEMENT

The authors declare no conflicts of interest.

## Data Availability

The data that support the findings of this study are available upon request from the corresponding author.
